# First-Principles Study of Halide Modulation on Deep-Level Traps in FAPbI_3_

**DOI:** 10.3390/nano15130981

**Published:** 2025-06-24

**Authors:** Jiaqi Dai, Wenchao Tang, Tingfeng Li, Cuiping Xu, Min Zhao, Peiqi Ji, Xiaolei Li, Fengming Zhang, Hongling Cai, Xiaoshan Wu

**Affiliations:** 1 National Laboratory of Solid State Microstructures, Department of Physics, Nanjing University, Nanjing 210093, China; 2 Institute of Materials Engineering, Nantong 226019, China

**Keywords:** halide perovskite, point defect, trap level, first-principle calculation

## Abstract

In this study, we investigate the influence of the halogen elements bromine (Br) and chlorine (Cl) on iodine defect properties primarily in FAPbI_3_ through first-principles calculations, aiming to understand the effect of high defect densities on the efficiency of organic–inorganic hybrid perovskite cells. The results indicate that Br and Cl interstitials minimally alter the overall band structure of FAPbI_3_ but significantly modify the defect energy levels. Br and Cl interstitials, with defect states closer to the valence band and lower formation energies, effectively convert deep-level traps induced by iodine interstitials (I_i_) into shallow-level traps. This conversion enhances carrier transport by reducing non-radiative recombination while preserving light absorption efficiency. Excess Br/Cl co-doping in FAPbI_3_ synthesis thereby suppresses non-radiative recombination and mitigates the detrimental effects of iodide-related defects.

## 1. Introduction

In recent years, solar cells based on halide perovskites, such as XPbI_3_ (X = Cs, CH_3_NH_3_, NH_2_CHNH_2_, etc.) have received widespread attention because of their advantages such as adjustable bandgap, high light absorption rate, low carrier recombination rate, and high photoelectric conversion efficiency (PCE) [1,2,3], which facilitate efficient charge collection and achieve higher PCE [4]. Due to these favorable optoelectronic properties, the PCE of single-junction perovskite solar cells (PSCs) has surged remarkably from 3.8% to over 26% within the past decade [5,6,7]. Among them, MAPbI_3_ (CH_3_NH_3_PbI_3_) and FAPbI_3_ (NH_2_CHNH_2_PbI_3_) have been the most studied. Compared with MAPbI_3_, FAPbI_3_ has a more symmetrical crystal structure and a more optimum theoretical bandgap of 1.48 eV [8], approaching the Shockley–Queisser limit for single-junction cells (approximately 33.7%) [5,6,9]. Moreover, FAPbI_3_ has better thermal stability and is not easily degraded [7]. Although the photoactive phase of FAPbI_3_ is unstable at operating temperatures and tends to transform into the photoinactive yellow phase [8], it remains a highly promising optoelectronic material because stabilization is achievable through doping with Zn (II) cations and formate anions, along with other strategies [10].

Despite rapid efficiency improvements, FAPbI_3_ faces a significant challenge in high intrinsic defect density. The soft lattice of synthesized halide perovskite solutions and films [5], combined with tilted FA cations within the relaxed PbI_6_ framework [11,12], induces strong lattice distortion that inevitably generates high defect concentrations [13,14,15]. While shallow defects dominate in halide perovskites [16], recent studies confirm the coexistence of detrimental deep-level traps in FAPbI_3_ [12,16,17,18]. The deep energy levels inside the bandgap usually have high ionization energy [19] and act as potent non-radiative recombination centers. By capturing charge carriers, they reduce carrier lifetimes, degrade open-circuit voltage (Voc), and ultimately limit device performance [20,21]. Consequently, even with advanced crystal growth techniques, FAPbI_3_ polycrystalline films exhibit high defect densities (9.6 × 10^15^~1.37 × 10^16^ cm^−3^) [22], exceeding those in classical semiconductors (typically 10^8^–10^15^ cm^−3^) [23,24,25,26] by orders of magnitude. This severely suppresses device efficiency. Although FAPbI_3_ exhibits some defect tolerance [27,28], strategies to mitigate defects remain crucial for further performance gains. The high defect density issue can currently be addressed through light soaking, chemical passivation, interface engineering, and microstructure modulation methods [29]. Recent studies demonstrate that doping and passivation with homologous halide ions effectively enhances halide perovskite performance. Mu et al. [30] found that 20% chlorine doping increases FAPbI_3_ power conversion efficiency. Energy-dispersive X-ray analysis confirms that the film’s chlorine contents match the precursor solution ratio, indicating precise chlorine doping improves FAPbI_3_ properties. Multi-halide co-doping shows significant performance optimization effects. Mariotti et al. [31] achieved 1.28 eV open-circuit voltage in single-junction cells and 32.5% certified tandem efficiency using triple-halide (I, Br, and Cl) perovskite with piperazine iodide interface engineering for perovskite–silicon tandem solar cells. Xu et al. [32] resolved phase separation through trihalide incorporation and established Br-mediated I-Cl lattice connections, realizing bulk chlorine doping that doubled carrier mobility and lifetime.

Current understanding remains limited regarding defect property modulation in halide perovskites through halide doping, and quantitative analyses of carrier transport and recombination effects are necessary. This study employs first-principles calculations to investigate intrinsic point defects in FAPbI_3_ and interstitial defect properties of bromine, chlorine, and fluorine. The calculations include defect formation energies, transition levels, and migration. The results show bromine and chlorine interstitial defects exert less influence on FAPbI_3_ performance than intrinsic iodine interstitials, accompanied by decreased migration rates. These defect property changes provide insights into FAPbI_3_ performance enhancement via halide doping.

## 2. Calculation Methods

This paper employs the Vienna ab initio Simulation Package (VASP 5. 3. 5), a computational software based on density functional theory [33]. The initial structure of FAPbI_3_ was constructed using experimentally reported perovskite geometries [34,35]. In examining the defect formation energy (DFE) of point defects in FAPbI_3_, a 2 × 2 × 2 supercell is employed (as shown in Figure S1), with a point defect formed into the supercell to facilitate the averaging of defect volume [36]. A plane–wave energy cutoff of 500 eV and a 5 × 5 × 5 k-point grid using the Monkhorst–Pack method were employed in the calculations. Structural relaxations were conducted until forces on atoms fell below 0.01 eV/Å, and energy convergence reached 1 × 10^−6^ eV. To account for van der Waals interactions, the rev-vdW-DF2 functional proposed by Hamada [37] was employed, ensuring full optimization of atomic positions and lattice parameters.

The effect of spin–orbit coupling (SOC) has been previously reported in Pb-based perovskites, but the predicted bandgap is significantly lower than the experimental value [38]. To obtain accurate bandgap values for qualitative analysis, we employed the G_0_W_0_ method with spin–orbit coupling (SOC) effects included [39,40], and the calculated bandgap is 1.50 eV, consistent with experimental values [41]. For the calculation of charged defects, it is assumed that the additional or missing electrons from charged defects come from an external electron reservoir or enter the supercell interior with their energy equivalent to the Fermi energy, using Equation (1) [42]:

(1) $$\mathrm{DFE}=E\left(\mathrm{defect}\right)-E(\mathrm{prefect})+\sum_{i}n_{i}\mu_{i}+q(E_{F}+E_{VBM})+E_{corr}$$

where E(defect) represents the system containing defects, E(prefect) is the energy of the perfect system without defects, and *n* and *μ* are the number of atoms and the corresponding chemical potentials individually. For vacancy defects, *n_i_* > 0, and interstitial defects, *n_i_* < 0. In order to ensure the formation of perovskite, the chemical potentials of FA, Pb, and I satisfy the following relationship:

(2) $$\mu_{FA}+\mu_{Pb}+3\mu_{I}=\Delta H\left({\mathrm{FAPbI}}_{3}\right)$$

Here, ΔH represents the formation energy of perovskite in the cubic phase. We selected the cubic phase of Pb solid, the orthorhombic phase of I_2_ solid, and the cubic phase of FA to calculate their chemical potentials, respectively. In order to prevent the formation of FAI and PbI_2_ as by-products, it is necessary to ensure that the chemical potential values satisfy the following conditions:

(3) $$\mu_{FA}+\mu_{I}<\Delta H(\mathrm{FAI})$$

(4) $$\mu_{Pb}+2\mu_{I}<\Delta H{(\mathrm{PbI}}_{2})$$

**Figure 1:**
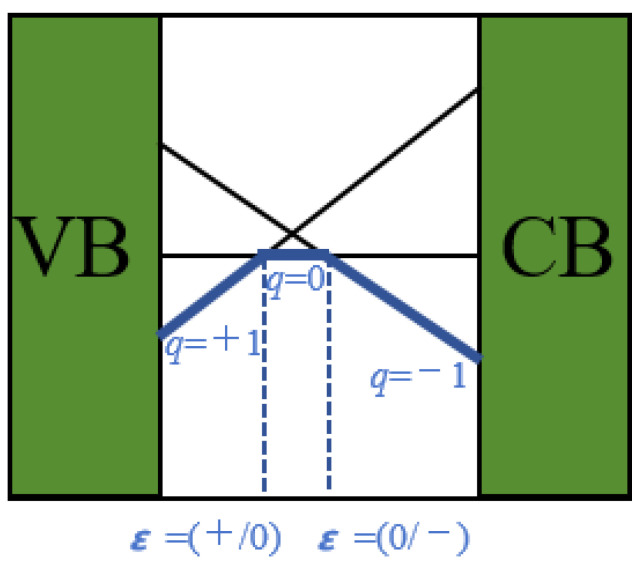
Schematic diagram of defect transition levels for defects in different charge states.

In accordance with Equations (2)–(4), the chemical potential regions under equilibrium growth conditions for the perovskite were calculated. In this research, the moderate chemical potentials of FA, Pb, and I were selected for calculating DFE (details of the chemical potential calculations are provided in Figure S2 and Table S1). In the last term $q(E_{F}+E_{VBM})+\Delta V$, *q* represents the charge of the defect; *E_VBM_* represents the energy level at the top of the valence band, and *E_F_* is the Fermi level at the top of its relative valence band. At the top of the valence band $E_{F}=0$, the Fermi levels are permitted to fluctuate within the bandgap, with the upper limit of the valence band maximum (VBM) aligning with the lower limit of the conduction band minimum (CBM) in a defect-free FAPbI_3_ crystal, which serves as the reference point for the bandgap. $E_{corr}$ is a correction term added to avoid spurious interactions of charged defects between supercells so that the potential energy in the defective supercells corresponds to the bulk potential energy (as shown in Supporting Information) [43,44]. Equation (1) shows that the formation energy of charged defects depends linearly on the Fermi level within the bandgap, with the slope determined by the charge state *q*. Consequently, defects of the same type can exhibit different formation energies for different charge states. The intersection points of these formation energy lines define the defect transition levels $\varepsilon (q_{1}/q_{2})$ between charge states [36]. Additionally, distinct charge states may correspond to distinct local atomic configurations [45,46,47]. Defect transition levels determine electronic behavior and are often used as a basis for experimental detection or identification of defects. Therefore, accurate calculation of the defect transition level is essential for the identification and characterization of defects. The specific calculation can be expressed by the following formula:

(5) $$\varepsilon (q_{1}/q_{2})=\frac{DFE^{q_{1}}-DFE^{q_{2}}}{q_{2}-q_{1}}$$

where $DFE^{q_{n}}$ is the DFE for a charge state of $q_{n}$. Figure 1 illustrates Equation (5). When the Fermi level lies below (+/0), the q = +1 charge state remains stable. When the Fermi level exceeds (+/0), the q = 0 state becomes stable. The (+/0) position defines the defect transition level (trap level). Similar principles apply to (0/−). The trap level position relative to band edges determines whether defects act as deep-level or shallow-level centers. Deep-level defects form when trap levels reside within the bandgap (mid-gap). These deep centers degrade carrier mobility [48].

## 3. Results and Discussion

**Figure 2:**
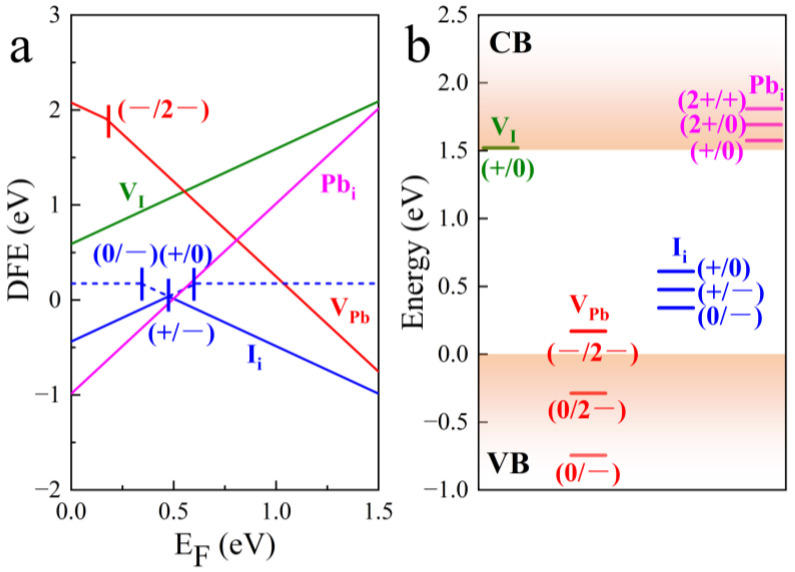
(**a**) Formation energies of stable defects within the bandgap, (**b**) trap levels of different defects.

Because organic cations in perovskite make little or no contribution to the electronic states at the edges of the energy bands, here, we ignore the effect of point defects associated with FA cations [39]. The defect formation energies of an iodine vacancy (V_I_), iodine interstitial (I_i_), lead vacancy (V_Pb_), and lead interstitial (Pb_i_) have been calculated and are shown in Figure 2a. The lowest formation energy defects are Pb_i_ and I_i_, which represent the fact that Pb_i_ and I_i_ are more likely to form and stabilize under thermodynamic equilibrium, implying a higher defect density. Stabilized I_i_ has a lower DFE. Figure 2a shows that I_i_ exists in neutral (0), negatively charged (−), and positively charged (+) states, with the stable charge state being the transition from + to −, forming a more stable negatively charged state. Similarly, Pb_i_ is also a relatively stable type of defect, with the stable charge state being 2+. In addition to these stable defects, the stable charge state for V_I_ within the bandgap is +, and for V_Pb_, it is −/2−, with the charge state reaching the lowest DFE being 2−. From the thermodynamic level, point defects are more prone to forming interstitial defects with lower DFEs. This phenomenon may be attributed to the soft lattice characteristics of FAPbI_3_, which facilitate the easier occupation of interstitial positions within the lattice by adding additional atoms [5]. While all defects will influence the position of the Fermi level, their effect will diminish exponentially with increasing DFE. The more stable I_i_ defect, for instance, affects the intrinsic Fermi level of the perovskite. In addition to considering the number of defects, it is also important to take into account their activity. This refers to the migration rate of the defects, as thermodynamically favorable defects may be limited by high migration activation energies, which prevent their migration. It is commonly accepted that interstitial defects are more prone to migration than vacancy defects [47], and defects associated with iodine exhibit a higher migration rate than those linked to lead [49,50]; thus, it can be reasonably deduced that defects related to iodine ions may become the primary migration targets [51,52,53]. Furthermore, defect transition levels (trap levels) critically influence charge carrier dynamics and photovoltaic performance in perovskite materials. Although some defects exhibit low defect formation energy, their impact on carrier transport depends primarily on trap level positions within the bandgap [54]. These trap levels act as non-radiative recombination centers, while charged defects reduce conductivity through carrier scattering. Crucially, halide perovskites demonstrate good defect tolerance because defect-induced trap levels typically reside near band edges, minimizing disruption to carrier transport.

Figure 2a shows the defect formation energy (DFE); under thermodynamic equilibrium, the stable charge states for V_Pb_ within the bandgap are −/2−, indicating that the defect can further capture holes through the trap levels shown in Figure 2b, releasing energy, hence the lower DFE for the 2− charge state. Since the neutral charge state of V_Pb_ is not stable under thermodynamic equilibrium, and the 0/2− and 0/− charge state transition levels are within the valence band, no deep-level traps are formed, meaning that the neutral charge state defect of V_Pb_ can be neglected. Furthermore, the 2− charge state migration activation energy of V_Pb_ indicates that metallic lead is only likely to nucleate on the surface under high temperatures or under illumination [55], so the impact of V_Pb_ on FAPbI_3_ is not significant. I_i_ can capture electrons and holes through the +/0 and 0/− trap levels at 0.89 eV below the conduction band (CB) and 0.34 eV above the valence band (VB), respectively, acting as both a donor and an acceptor within the bandgap. The stable trap levels at the thermodynamic level, as illustrated in Figure 2a, are in close proximity to the Fermi level of FAPbI_3_. This indicates that the trap level exerts a considerable influence on the electronic states situated in proximity to the Fermi surface. Thus, FAPbI_3_ performance degrades significantly because all trap levels reside within the bandgap, and deep-level traps actively capture carriers, reducing carrier lifetime. As shown in Figure 2b, all trap levels of Pb_i_ and V_I_ are located inside the CB, and there are no trap levels inside the bandgap, which makes it difficult to form a recombination center and has less effect on the carrier transport. Moreover, the +/0 trap level of V_I_ and Pb_i_ show shallow trapping behavior at the bottom of the CB, with some polarization around the defect location, and have a resonance with the CB, so that despite the migration activation energy being low, it can still act as a shallow-level trap [56]. From the above conclusion, I_i_ defects can be considered as the most likely point defect to exist stably and trap carriers because they have the lowest DFE with deep-level traps.

**Figure 3:**
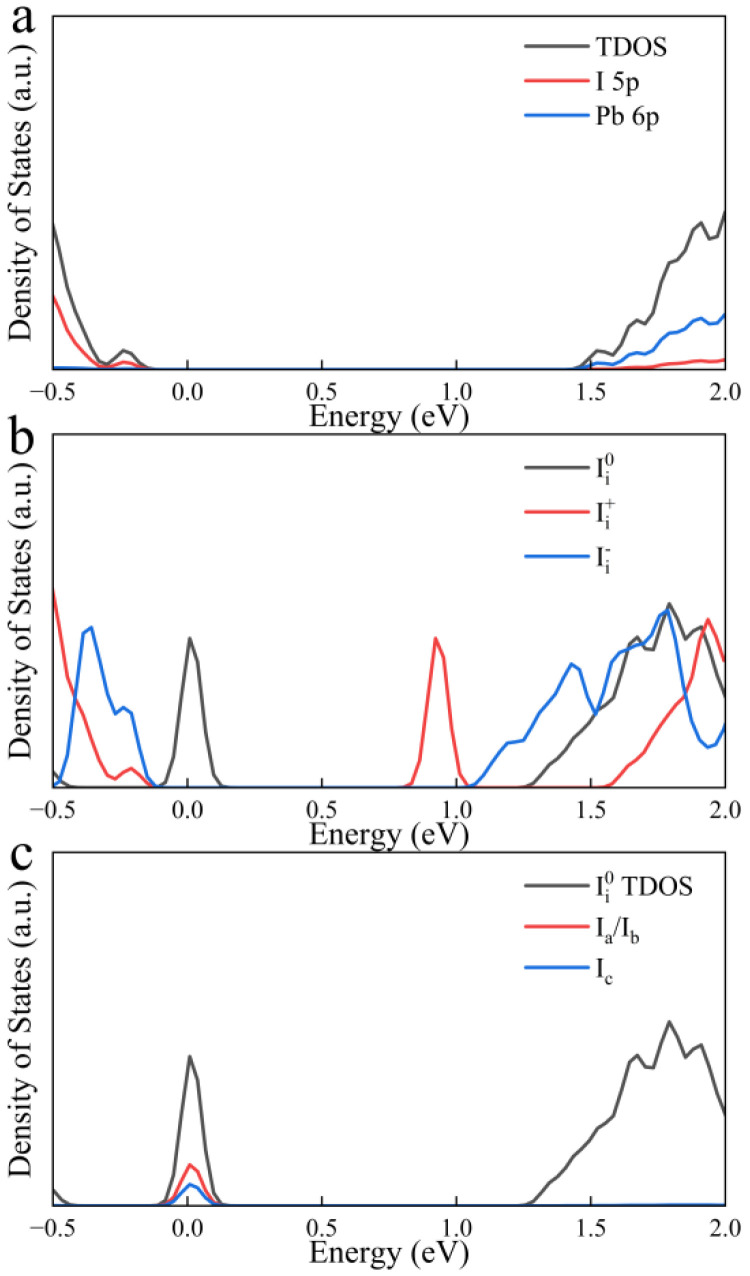
(**a**) Density of states of FAPbI_3_ without defects and I 5p and Pb 6p orbitals, (**b**) Density of states of the three charge states of I_i_, (**c**) Partial density of the interstitial atoms I_c_ and adjacent atoms I_a_/I_b_.

To further investigate the effect of I_i_ on FAPbI_3_, we calculated the density of states in the case of I_i_, and the density of states calculation from Figure 3a shows that the conduction band of FAPbI_3_ is mainly contributed by electrons in the 6p orbital of Pb atoms, while the valence band is mainly contributed by electrons in the 5p orbital of I atoms. Other elements introduce deep-level states within the VB and CB. These states do not participate in bonding and contribute negligibly to the density of states near the band edges.

As shown in Figure 3b, the different charge states of I_i_ form traps inside the bandgap with different energy level positions, $I_{i}^{0}$ creates a new trap level within the bandgap that can trap either holes or electrons, but according to the Pauli exclusion principle, these two processes cannot occur at the same time, and since its location is closer to the VB, hole trapping can become a dominant process, and as more holes are trapped, the holes that accumulate in the trap level can trap a large number of photo-excited electrons in the valence band, making it more difficult for valence band electrons to transition to the conduction band, reducing the number of electrons that can be collected by the electron transport layer. It is worth noting that due to the close proximity of the trap level location to the VBM if the defect concentration is too high, Fermi level pinning will form, which further enhances non-radiative recombination of electrons and holes and reduces carrier concentration, which consequently decreases both open-circuit voltage and short-circuit current, and induces more pronounced hysteresis [57,58,59]. By observing the $I_{i}^{0}$ DOS of Figure 3c, it is found that the interstitial I atom has the same peak position of state density with its neighboring I atoms b and c, indicating that I ions adjacent to this interstitial atom contribute to the formation of the defect state $I_{i}^{-}$ does not create new trap levels inside the bandgap. This phenomenon arises because the interstitial iodine anion forms an I-Pb bond with the lead ion, which eliminates the unsaturated suspension bond and moves the trap state into the VB, which helps to eliminate the effect of electron–phonon coupling on the lattice.

In the $I_{i}^{+}$ system, the trap level formed by positively charged iodine cations is located below the CBM, and this trap state can act as an electron acceptor to capture the electrons of valence band transitions, forming electron traps and accelerating non-radiative recombination. In addition, the positively charged interstitial iodide cations are prone to form I trimers by mutual attraction with the point anions on the lattice, which leads to an increase in the number of trapping sites and will result in the localization of charge density to itself, thus reducing the charge density of the VBM and CBM.

The results from previous molecular dynamics simulations suggest that trap levels can be eliminated and carrier lifetimes extended by oxidizing $I_{i}^{0}$ to $I_{i}^{-}$ [60]. However, $I_{i}^{-}$ is unstable under light conditions, and holes are easily captured and converted back to $I_{i}^{0}$ again, reducing the performance of perovskite [48,61]. The preceding analysis indicates that the impact of I_i_ on the electronic structure of perovskite is more pronounced among the point defects.

**Figure 4:**
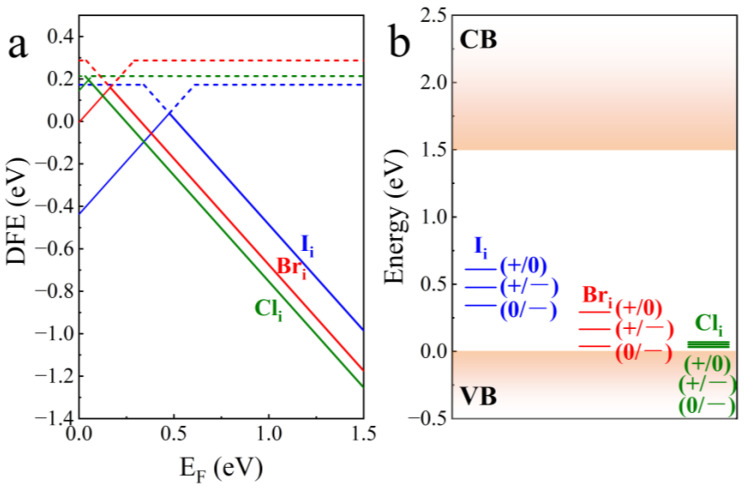
(**a**) Formation energies of halide (I, Br, and Cl) interstitial defects within the bandgap, (**b**) Trap levels of halide (I, Br, and Cl) interstitial defects.

In order to minimize the effect of I_i_ on FAPbI_3_, based on the same model, we investigated the possible effect of substitution of I_i_ by the halides Br_i_ and Cl_i_ on FAPbI_3_. From the DFE of Figure 4a, it can be seen that the negative charge states (−) of Br_i_ and Cl_i_, which are stabilized inside the bandgap at the thermodynamic level, are the most stable, which is the same as that of I_i_, and the magnitude of the DFE is I_i_ > Br_i_ > Cl_i_, which represents the fact that Br_i_ and Cl_i_ are more likely to form, this may be due to the fact that Cl and Br with smaller ionic radii than I are more likely to form interstitial defects that do not cause excessive distortion stresses on the lattice leading to elastic distortions, and therefore require less energy cost and are more likely to occupy interstitial positions in the lattice, and these smaller ionic radii may be more likely to chemically bond with neighboring atoms or ions, resulting in stable chemical bonding with fewer dangling bond effects on the defect state [62]. Compared with I_i_, the trap levels of Br_i_ and Cl_i_ demonstrated in Figure 4b become more concentrated, the transition levels of defects between different charge states occur more easily with less energy change required, and the trap level as a whole is significantly more biased towards the valence band, gradually shifting from deep-level traps inside the bandgap to shallow-level traps, implying that electrons or holes captured by the trap state can be easily ionized by smaller ionization energies. This renders it difficult to form recombination centers, which reduces the impact on carrier transport, consistent with the improved photovoltaic performance reported in previous studies [63].

We also calculated defect formation energy for F_i_ replacing I_i_, as shown in Figure S3a. The defect formation energy of F_i_ is significantly higher than that of I_i_, Br_i_, and Cl_i_. This indicates that F_i_ formation in FAPbI_3_ is energetically unfavorable, making it difficult for F_i_ to occupy I_i_ sites. Additionally, fluorine demonstrates inferior trap level modulation capability compared with bromine and iodine in Figure S3b. Fluorine ions likely exhibit minimal impact on regulating deep-level traps in FAPbI_3_. Subsequent discussions will focus on Br_i_ and Cl_i_ properties.

**Figure 5:**
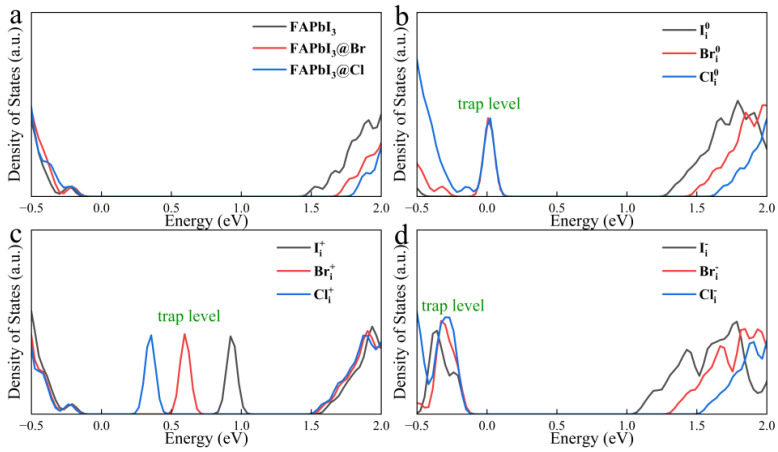
(**a**) Density of states of Br and Cl occupying the I position inside the lattice, (**b**) Density of states of interstitial defects at neutral charge state, (**c**) Density of states of charge defects at positive charge (+) state, (**d**) Density of states of charge defects at negative charge (−) state.

Given the low formation energies of Br_i_ and Cl_i_ and their lack of deep-level traps, we investigated their electronic density of states (DOS). Figure 5a reveals that substituting lattice iodine with bromine or chlorine introduces no in-gap states and only slightly widens the bandgap. This suggests minimal disruption to the fundamental electronic structure of FAPbI_3_ and largely preserved electronic transport properties. From Figure 5b, it can be found that at a charge state of 0, compared with $I_{i}^{0}$, ${\mathrm{Br}}_{i}^{0}$, and ${\mathrm{Cl}}_{i}^{0}$ make the positions of VB and CB shifted upwards as a whole, which causes the positions of the trap levels to be progressively closer to the valence band, which is more susceptible to ionization. The trap levels formed by the positive charge defects in Figure 5c are located deep in the bandgap, and due to the +/− charge state transitions, there is a trapping behavior of electrons. The positively charged defects act as electron acceptors, and the trap level of $I_{i}^{+}$ is close to CB; thus, it is expected that the trapping of the electrons will take place much more rapidly, whereas the trapping of electrons becomes more and more difficult in the case of ${\mathrm{Br}}_{i}^{+}$ and ${\mathrm{Cl}}_{i}^{+}$ because of the gradual location of trap levels closer to the VB, it is notable that the positively charged defects, Br_i_ and Cl_i_, are not the lowest energy of formation at the thermodynamic level. Consequently, the density of these defects is not expected to be particularly high.

The reduced formation energies of Br_i_ and Cl_i_ suppress the generation of I_i_ defects. Compared with the deep-level traps formed by I_i_, shallow-level traps exert less detrimental effects on carrier transport, which may benefit the performance of FAPbI_3_. This suggests that Br/Cl doping effectively inhibits non-radiative recombination and prolongs carrier lifetime in halide perovskites, consistent with recent experimental studies. For instance, Zhang et al. [64] used synchrotron-based XRD and XPS to demonstrate that Cl-terminated ligands optimize halogen distribution in FAPbBr_3-x_Cl_x_ nanocrystalline films. This optimization induces a 9 nm blue shift in the PL peak and a 1.6-fold increase in exciton binding energy, confirming reduced deep-level traps. Similarly, Br-rich surfaces in CsPbBr_3_ nanosheets significantly enhance the PLQY (from 31.15% to 87.2%) and extend carrier lifetime (to 16.69 ns), as reported by Varnakavi et al. [65]. These improvements further evidence the defect-passivation effect of Br doping.

**Figure 6:**
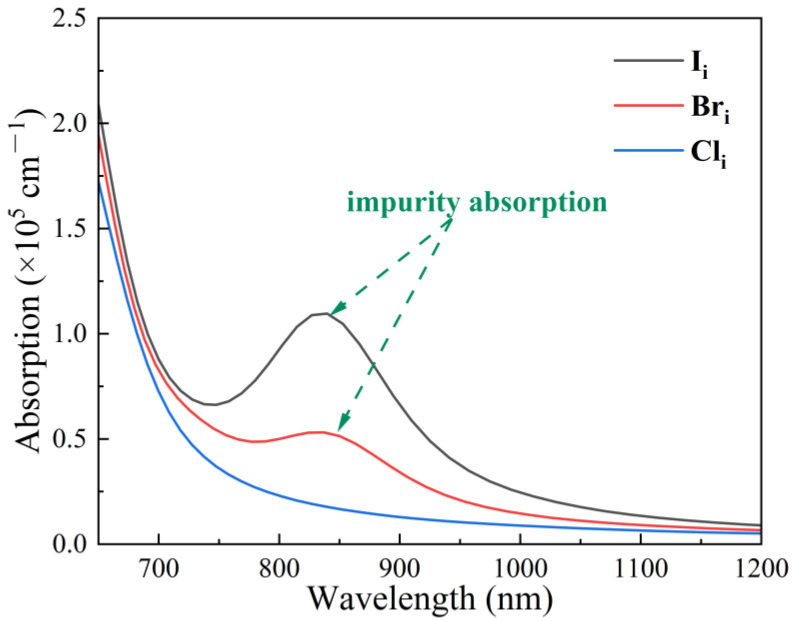
FAPbI_3_ with halide (I, Br, and Cl) interstitial defects optical absorption spectra.

The aforementioned conclusions lead to the conclusion that Br_i_ and Cl_i_ primarily influence defect properties rather than the fundamental electronic structure of FAPbI_3_. To further investigate their capacity to modulate I_i_ defects, we examined the impact of the defects on the nature of light absorption. Figure 6 illustrates the optical absorption coefficient versus wavelength for FAPbI_3_ with I_i_, Br_i_, and Cl_i_ interstitial defects. These results demonstrate that the intrinsic absorption of FAPbI_3_ remains nearly identical regardless of I_i_, Br_i_, or Cl_i_ interstitial defects, confirming consistent optical bandgaps. The absorption observed in long-wavelength regions originates from FAPbI_3_ containing Ii defects, indicating defect-mediated absorption.

The absorption edge that emerges after adding impurities to the absorption peak suggests that the defect is fully ionized. The impurity absorption position of Br_i_ is slightly blue-shifted, and the absorption peak is diminished, which suggests that Br_i_ trap level necessitates less ionization energy. Additionally, Cl_i_ exhibits negligible impurity absorption because of its shallow trap levels, consistent with its rapid ionization behavior., which corresponds to the previous Figure 5b, the shallow-level trap of Cl_i_ is readily ionized. The suppression of the trap level by Br and Cl has been demonstrated to effectively reduce the effect of I on the light-absorbing properties of FAPbI_3_, as evidenced by the absorption spectra.

**Figure 7:**
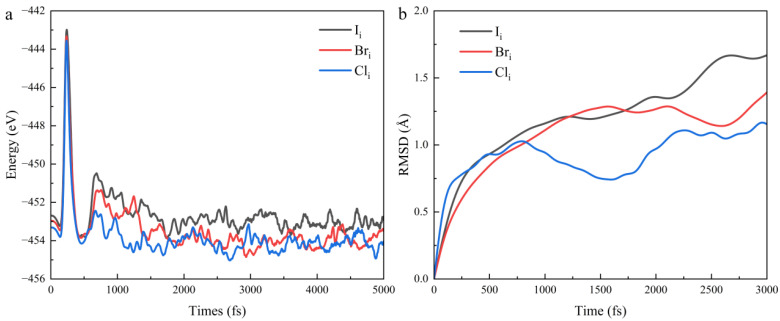
(**a**) Energy evolution of FAPbI_3_ containing different halide (I, Br, and Cl) interstitial defects, (**b**) Root mean square displacement variations.

To evaluate the dynamic behavior of different interstitial defects (I_i_, Br_i_, and Cl_i_), we performed molecular dynamics (MD) simulations at 300 K using the NVT ensemble with a Langevin thermostat [66,67]. The simulations utilized a time step of 1 fs and spanned 5 ps after equilibration. As shown in Figure 7a, the energy fluctuations of the three supercells are within a narrow range, and no significant atomic rearrangements or phase transitions occur during the simulation. Consequently, we conclude that FAPbI_3_ containing halide defects is generally thermally stable [68]. Figure 7b shows the root mean square displacement (RMSD) of interstitial atoms versus time. To determine the RMSD in the equilibrium state, the first 2 ps were excluded as the pre-equilibration period. We can observe that the RMSD follows the trend of I_i_ > Br_i_ > Cl_i_. This indicates that interstitial iodine atoms exhibit larger displacements, suggesting higher dynamic mobility compared with bromine and chlorine interstitials [69]. This may make interstitial defects more prone to ion migration and defect recombination, limiting the efficiency and stability of FAPbI_3_ [70]. For this purpose, we calculated the diffusion coefficients using root mean square displacement, and these values are presented in Table 1 (see Supporting Information for calculation details).

The calculation results we obtained match those reported in previous studies [51,71]. Through the analysis of diffusion coefficients, it has been demonstrated that I_i_ exhibit significantly higher diffusivity compared with Br_i_ and Cl_i_, indicating that I_i_ possess the fastest migration rates within the crystal lattice, which exerts a more significant influence on the performance and stability of FAPbI_3_ perovskite compared with Br_i_ and Cl_i_.

The above calculations show that Br_i_ and Cl_i_ can reduce the impact on the performance of perovskite solar cells by transforming the deep-level traps formed by I_i_ into shallow-level traps, thus improving the performance of FAPbI_3_. In a previous study, Lyu et al. [72] added FAX additives (X = Br or Cl) to a solution of perovskite precursor and characterized the properties of FAPbI_3_ after treatment with the additive. It was observed that the perovskite solar cells exhibited an enhancement in the short-circuit current density (J_sc_), open-circuit voltage (V_oc_), and PCE following the addition of FABr and FACl into the FAPbI_3_ precursor solution. This indicates that the incorporation of Cl and Br ions markedly enhances the carrier lifetime, with the Cl ion modifier exhibiting a more pronounced impact, which aligns with the calculated outcomes [73,74].

We further investigated the performance of FAPbI_3_ cells using Br and Cl as additives (Table 2). These data show that halide additives significantly enhance the photoelectric conversion efficiency (PCE) and improve the long-term stability of FAPbI3. This improvement is consistent with the ability of halides to modulate defects, as identified in this study. This reflects the potential of Br and Cl doping or addition in the preparation of perovskites free of cations, such as MA^+^ and Cs^+^.

## 4. Conclusions

This study employs first-principles calculations to investigate halide doping effects on intrinsic point defects in FAPbI_3_. The results show that the low formation energies of Br_i_ and Cl_i_ transform deep-level traps from I_i_ into shallow-level traps based on Br_i_ and Cl_i_. Molecular dynamics calculations reveal I_i_ defects diffuse more readily than Br_i_ or Cl_i_. With minimal bandgap changes, halide defects (Br_i_ or Cl_i_) mitigate detrimental effects on perovskite device performance compared with intrinsic FAPbI_3_ defects. This explains the mechanism behind FAPbI_3_ performance enhancement via halide doping. Future work should systematically study halide doping effects on FAPbI_3_ carrier transport properties and develop co-doping strategies.

## Figures and Tables

**Table 1:** Calculated diffusion coefficient on different interstitial defects.

Defect	Diffusion Coefficient cm^2^·s^−1^
I_i_	4.53 × 10^−8^
Br_i_	3.24 × 10^−8^
Cl_i_	2.67 × 10^−8^

**Table 2:** Performance improvement of FAPbI_3_ with Br or Cl as dopant or additive.

Device Architecture	Solution	PCE Before Solution %	PCE After Solution %	Stability Performance	Ref.
FTO/SnO_2_/FAPbI_3_/Spiro-MeOTAD/Au	FACl/FABr additive	16.55	22.51/20.08	-	[72]
FTO/TiO_2_/FAPbI_2.8_Cl_0.2_/Spiro-MeOTAD/Au	FACl dopant	15.7	17.0	-	[30]
FTO/TiO_2_/FAPbI_2.9_Br_0.1_/Spiro-MeOTAD/Au	FAPbBr_3_ dopant	12.98	16.569	-	[75]
FTO/SnO_2_/FAPbI_3_/Spiro-MeOTAD/Ag	PACl additive	17.39	21.45	40 °C, 40%RH, AM 1.5 G, 70% PCE after 140 h.	[76]
FTO/SnO_2_/FAPbI_3_/Spiro-MeOTAD/Au	PFACl additive	22.8	24.4	60 °C, 25 ± 5%RH, 85% PCE after 500 h.	[77]
ITO/SnO_2_/FAPI_3_/Spiro-MeOTAD/Au	FFEACl additive	23.04	25.41	25 °C, 30%RH, AM 1.5 G, 83% PCE after 1000 h.	[78]
ITO/PTAA/FAPbI_3_/C_60_/BCP/Ag	FABr additive	11.31	14.38	-	[79]
FTO/TiO_2_/FAPbI_3_/Spiro-MeOTAD/Au	FACl + FAAC additive	16.42	20.92	50 °C in N_2_, 87% PCE after 1628 h.	[80]
ITO/PCBM/FAMAPbI_3-X_Br_X_/P3HT/Ag	FACl additive	18.60	21.02	25–35 °C, 10–25%, 90% PCE after 1200 h.	[81]

## Data Availability

Data are contained within the article and Supplementary Materials.

## Supplementary Materials

The following supporting information can be downloaded at https://www.mdpi.com/article/10.3390/nano15130981/s1. Figure S1. (a) Interstitial defects (red atoms represent I, Br, Cl or F interstitial atoms); (b) lead vacancy defects, and (c) iodine vacancy defects in a 2×2×2 FAPbI_3_ super cell. Figure S2. The thermodynamically stable range for equilibrium growth of FAbI_3_ is a narrow yet elongated region marked in red. Figure S3. (a) Formation energies of stable defects within the bandgap, (b) trap-levels of different defects. Table S1. The allowed chemical potential values for perovskite growth under thermal equilibrium conditions were calculated, taking into account three constraints.
