# Supplementary material for: First-Principles Study of Halide Modulation on Deep-Level Traps in FAPbI3
Source: Nanomaterials (Basel). 2025 Jun 24;15(13):981. doi: 10.3390/nano15130981 (PMC12251310; doi:10.3390/nano15130981)
Supplement: Supplementary file 1 [file nanomaterials-15-00981-s001.zip › nanomaterials-3665227-supplementary.pdf]

Supporting Information

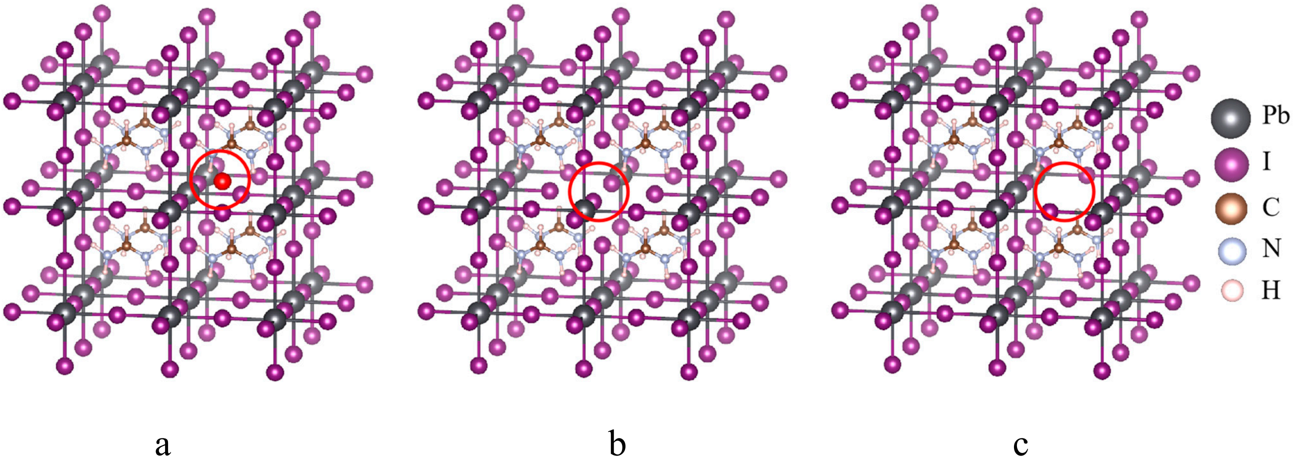

Figure S1. (a) Interstitial defects (red atoms represent I, Br, Cl or F interstitial atoms); (b) lead vacancy defects, and (c) iodine vacancy defects in a 2×2×2 $FAPbI_3$ super cell.

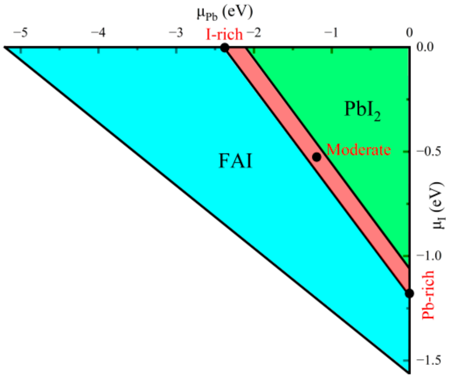

Figure S2. The thermodynamically stable range for equilibrium growth of $FAbI_3$ is a narrow yet elongated region marked in red.

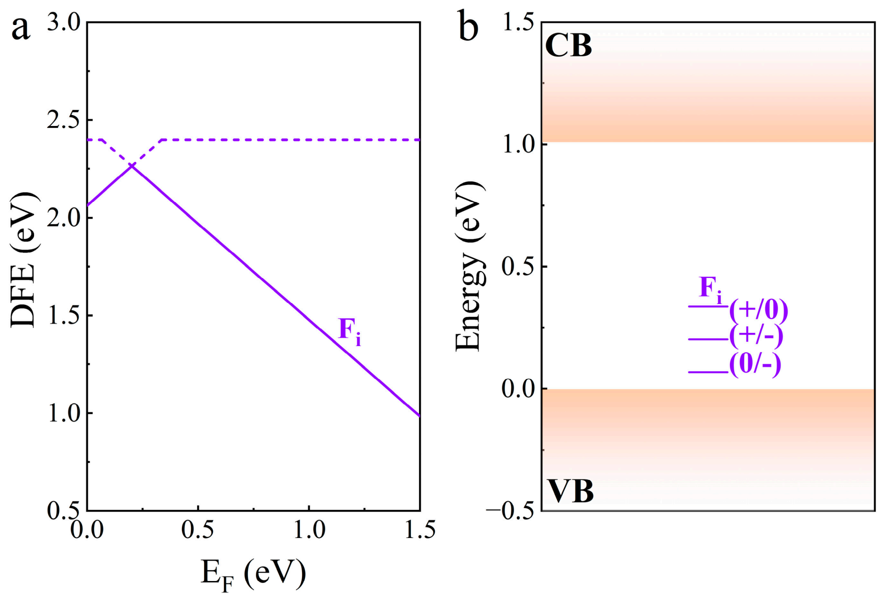

Figure S3. (a) Formation energies of stable defects within the bandgap, (b) trap-levels of different defects.

Table S1. The allowed chemical potential values for perovskite growth under thermal equilibrium conditions were calculated, taking into account three constraints.

	Pb-rich	I-rich	Moderate
μ_{FA}	-1.58	-2.61	-2.08
μ_{Pb}	0.00	-2.40	-1.20
μ_{I}	-1.16	0.00	-0.58

1.1 Energy Corrections

The Freysoldt-Neugebauer-van de Walle (FNV) correction scheme was employed to address the finite-size effects appearing in equation 1 [1,2].

$$E_{corr} = E_q^{latt} - q(\Delta V_{0/p} + \Delta V_{q-0/m}) \tag{S.1}$$

The first term E_q^{latt} in Eq. (S.1) is the lattice energy correction at the qth charge-state.

$$E_q^{latt} = E_q^{iso,m} - E_q^{per,m} \tag{S.2}$$

$$\Delta V_{0/p} = V_0 |_{far} - V_{pr} \tag{S.3}$$

$$\Delta V_{q-0/m} = [V_q - V_0]_{far} - V_q^{per,m} |_{far} \tag{S.4}$$

Here, $E_q^{iso,m}$ represents the model lattice energy of an isolated defect in the dielectric medium, while $E_q^{per,m}$ denotes the model energy of the defect in the periodic structure. Where $V_0|_{far}$ is the potential value of the neutral defect far from the defect position and V_{pr} is the potential of the pristine material. $\Delta V_{q-0/m}$ is the potential difference $[V_q - V_0]_{far}$ of the charged defect relative to the model periodic lattic potential $V_q^{per,m}|_{far}$ far from the defect. The lattice energy correction terms were computed utilizing the Coffee software package [3].

1.2 Diffusion coefficient and ionic mobility

The mean square displacement (MSD) is defined as the average of the square of the displacement of a particle over a given time interval. Its direct relationship with the diffusion coefficient D is governed by the Einstein relation [4]:

$$D = \frac{1}{6} \lim_{\Delta t \to \infty} \frac{d(\mathrm{MSD})}{d\Delta t} \tag{S.5}$$

When the MSD exhibits linear growth with time (in the diffusive regime), the slope corresponds to 6D, i.e., $\mathrm{MSD} = 6D\Delta t$.

References

1. Freysoldt, C.; Neugebauer, J.; Van de Walle, C.G. Fully ab initio finite-size corrections for charged-defect supercell calculations. *Phys. Rev. Lett.* **2009**, *102*, 016402, doi:10.1103/PhysRevLett.102.016402.
2. Komsa, H.P.; Pasquarello, A. Finite-size supercell correction for charged defects at surfaces and interfaces. *Phys. Rev. Lett.* **2013**, *110*, 095505, doi:10.1103/PhysRevLett.110.095505.
3. Naik, M.H.; Jain, M. CoFFEE: Corrections For Formation Energy and Eigenvalues for charged defect simulations. *Comput. Phys. Commun.* **2018**, *226*, 114-126, doi:10.1016/j.cpc.2018.01.011.
4. Woolard, E.W.; Einstein, A.; Furth, R.; Cowper, A.D. Investigations on the Theory of the Brownian Movement. *The American Mathematical Monthly* **1928**, *35*, doi:10.2307/2298685.
